# Effects of High-Impact Exercise on Bone Marker Concentrations During Controlled Low Energy Availability in Recreational Female Runners

**DOI:** 10.1007/s00223-026-01564-0

**Published:** 2026-06-25

**Authors:** Trisha Sterringer, Craig Sale, Anna Morozov, Jiarui Liang, D. Enette Larson-Meyer

**Affiliations:** 1 Virginia Tech, Blacksburg, VA USA; 2 Manchester Metropolitan University, Manchester, UK

**Keywords:** Female, Endurance, Markers of bone remodeling, Nutrition, Recreational athlete

## Abstract

Exposure to low energy availability (LEA) can stimulate metabolic adaptations that have been shown to disrupt bone remodeling as suggested by suppressed markers of bone formation and/or increased markers of bone resorption. Previous studies suggest that brief, high-impact exercise during short-term energy restriction may stimulate an osteogenic response and mitigate the negative effect of LEA on bone health. This randomized, crossover study of twelve recreational female runners (age: 26.4 ± 3.9 years, VO_2max_: 45.3 ± 7.2 mL/kg/min) assessed the short-term effects of jumping exercises on markers of bone formation and resorption after five days of experimentally-controlled LEA (15 kcal/kgFFM/d). Participants completed two LEA conditions that involved daily treadmill running with either no additional exercise (RUN) or an additional 50 jump exercises per day (RUN + J). Fasted bone markers and hormone concentrations were measured on the mornings immediately before and after each intervention period. Bone resorption marker β-CTX was increased over time in both LEA conditions (main effect of time: *P* = 0.005; preRUN: 0.32 ± 0.21, postRUN: 0.36 ± 0.15 ng/mL; preRUN + J: 0.38 ± 0.21, postRUN + J: 0.42 ± 0.19 ng/mL) with no interaction effect (*P* = 0.716). Bone formation biomarkers, PINP and sclerostin, did not change over time (*P* = 0.517, *P* = 0.091) or between conditions (*P* = 0.187, *P* = 0.666). In conclusion, performing 50 jumping exercises during short-term LEA did not have an effect on markers of bone resorption or formation during short-term LEA induced by running exercise and dietary energy restriction.

**Supplementary Information:**

The online version contains supplementary material available at 10.1007/s00223-026-01564-0.

## Introduction

Low energy availability (LEA) is a concern for athletes and highly active individuals, as it can contribute to impairments in health and sports performance [1]. Competitive and recreational athletes experience LEA when energy intake (EI) is insufficient to support optimal physiological function after accounting for exercise energy expenditure (EEE). Energy availability (EA) of < 30 kcal per kilogram of fat-free mass per day (kcal/kgFFM/d) is commonly used as the threshold for LEA in female athletes [2]; however, LEA risk is also identified using validated screening tools [3] and objective measures of endocrine and/or metabolic perturbations [4]. The prevalence of LEA risk (measured by validated questionnaire) and symptoms associated with LEA amongst runners is typically higher in females compared to males [5], with an estimated 19 to 60% of recreational female runners considered ‘at risk’ for LEA when assessed using the Low Energy In Females Questionnaire (LEAF-Q; score > 8) [6, 7].

Exposure to LEA may be intentional (e.g., dietary restriction) or inadvertent (e.g., lacking knowledge of energy needs during training, decreased appetite, food insecurity) and corrected by adjusting EI and/or EEE. However, some athletes may be hesitant to correct LEA if they are intentionally trying to manipulate body composition or body weight. Although controlled periods of LEA may not hurt athletic performance acutely, experimentally-induced LEA has been shown to alter physiological processes in the first few days of exposure, evidenced by changes in hormone concentrations, metabolic rate, and markers of bone (re)modeling [8–11]. Concern has been raised about the lack of longitudinal data on athletes exposed to repeated periods of LEA (e.g., weight cycling in weight-sensitive sports, energy deficits during high training periods) throughout athletic careers, given the potential impact it may have on long-term bone health [12, 13].

Biomarkers of bone formation and resorption are useful when assessing the short-term effects of LEA on bone metabolism because it can take months or years for measurable changes in bone density and architecture to occur. The recommended reference markers for bone formation and resorption are procollagen type 1 N-terminal propeptide (P1NP) and β-carboxyterminal telopeptide of type 1 collagen (β-CTX), which are byproducts of type 1 collagen synthesis and degradation, respectively [14]. Over 90% of the organic bone matrix is comprised of type I collagen which is synthesized from procollagen type 1 by osteoblasts; however, type 1 collagen is not specific to bone and is expressed in skin, blood vessels, tendons, and other connective tissues [15]. Previous controlled LEA studies in females have shown increased concentrations of β-CTX and suppressed P1NP in as little as three to five days when EA is reduced to less than 20 kcal/kgFFM/d [8, 16, 17]. There could be a potential risk for decreased bone accrual, strength, and bone mineral density (BMD) if the long-term rate of bone formation is insufficient relative to the rate of resorption over time. In adult women, lifetime history of fasting and restrictive eating disorder behaviors has been associated with lower total body and hip BMD in mid-life [18]. Thus, athletes exhibiting restrictive eating behaviors during their athletic careers may be at risk for impaired bone health later in life.

In the absence of energy deficiency or overtraining, weight-bearing exercise generally has an anabolic effect on bone by stimulating the (re)modeling cycle and synthesis of new bone tissue [19]. Engaging in high-impact jumping has even been shown to attenuate LEA-induced increases in bone resorption of females during short-term LEA without any additional activity [17]. This suggests that high-impact exercise may counteract some of the negative consequences of LEA in the absence of other weight-bearing activity; however, it is unknown if these protective effects persist when combined with running. Therefore, the purpose of this study was to evaluate the short-term effects of high-impact jumping exercises on bone resorption and formation markers in female runners during a controlled period of LEA compared to a period of controlled LEA without jumping. A secondary aim was to evaluate the responses of bone-regulatory hormones between conditions.

## Methods

### Overview of Study Design

**Fig. 1:**
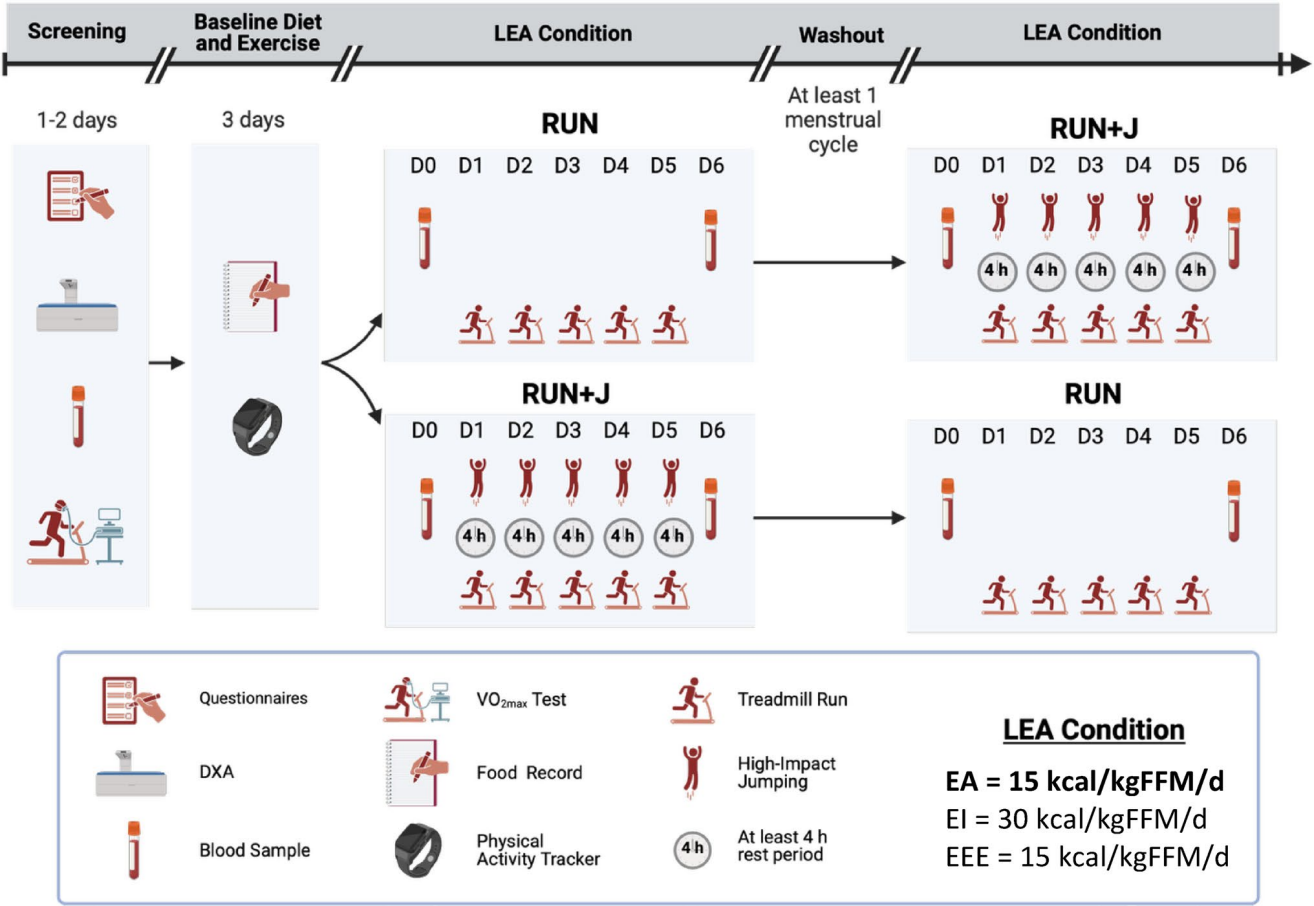
Overview of crossover study design. During screening, participants completed health questionnaires, dual-energy x-ray absorptiometry scan for body composition assessment, blood sample collection, and VO_2max_ test. Baseline diet and exercise were assessed using three-day food records and a wearable physical activity tracker. Participants were then randomized to the RUN or RUN + J condition. There was a washout period of at least one menstrual cycle between experimental conditions. Abbreviations: DXA, dual-energy x-ray absorptiometry; LEA, low energy availability; EI, energy intake; EEE, exercise energy expenditure. Figure created with BioRender.com

This study employed a randomized crossover design to investigate the short-term effect of high-impact jumping exercises on bone resorption and formation in recreational female runners during controlled LEA. A computer-based random number generator for research (randomizer.org, Version 4.0) [20] was used to randomize the participants into the condition order. Participants completed two 5-day experimental conditions separated by at least one menstrual cycle. EA was controlled at 15 kcal/kgFFM/d through dietary restriction (EI = 30 kcal/kgFFM/d) and daily exercise (EEE = 15 kcal/kgFFM/d) consisting of treadmill running at 65–70% VO_2max_ either with additional jumping exercises (RUN + J) or without (RUN). Experimental conditions began during the follicular phase of the menstrual cycle, one to seven days after the onset of menses (Fig. 1).

Study eligibility and aerobic fitness were assessed during two screening visits, followed by three days of normal diet and exercise tracking using written food diaries and exercise records. Participants notified the research team at the onset of menstruation, and the intervention began within the following seven days based on the participants’ and laboratory availability. Participants who could not be scheduled within seven days of menstruation were delayed until the following menstrual cycle. Fasting blood samples were collected on the mornings immediately before and after each intervention period. Compliance with the nutrition and exercise intervention was evaluated daily by collecting all food packaging and uneaten items, daily measurement of body mass, and physical activity data collected by a wearable smartwatch (Venu SQ, Garmin International, Olathe, KS, USA). This study was approved by the Virginia Tech Institutional Research Board (IRB #22-168), and participants provided written informed consent before participating.

### Participants

Sixteen recreational female runners were recruited from the local area via flyers and digital communications. Eligible participants were between 18 and 35 years of age with self-reported normal menstrual cycles (24 to 32 days), a body mass index (BMI) between 18.5 and 30.0 kg/m^2^, and were non-smokers, not using hormonal contraceptives, not pregnant or lactating, and were free from bone injury within the previous 6 months. Individuals with low bone density (z-score < -2), low hemoglobin (< 12.0 g/dL), abnormal thyroid function (thyroid stimulating hormone; TSH < 0.4 mIU/L or > 5.5 mIU/L) [21], risk of existing LEA (LEAF-Q score > 8), and those who had recently recovered from an eating disorder within the last 12 months, or used medications that would affect study results (e.g., corticosteroids, anticonvulsants, gonadotropin-releasing hormone agonists) were excluded. Eligibility was confirmed using a standardized health history questionnaire, urine pregnancy test, blood biochemistry, and body composition assessment by dual-energy x-ray absorptiometry (DXA) during the initial screening visit. Participants were classified according to the participant classification framework proposed by McKay and colleagues [22] using self-reported training habits and level of competition.

### Screening

Participants reported to the laboratory for the initial screening visit in a rested state after a 10-h overnight fast. Anthropometric and body composition measurements were performed with participants dressed in lab-provided standardized clothing (t-shirt and shorts), without jewelry, and after voiding their bladders. Height was measured to the nearest 0.1 cm using a stadiometer (Welch Allyn Scale-Tronix 5002, Milwaukee, WI, USA), and body mass was measured to the nearest 0.1 kg on a digital physician’s scale (WB-110A NETP III, Tanita Corporation of America, Arlington Heights, Illinois, USA). Fat mass (FM), FFM, lean body mass (LBM), body fat percentage (BF%), and BMD were assessed using a fan-bean DXA (Lunar iDXA, enCORE Version 15, General Electric Healthcare, Madison, WI, USA). The DXA scanner was calibrated daily according to the manufacturer’s instructions. Eleven of the twelve DXA scans were performed and analyzed by the same licensed DXA technician (TS). Participants were in a supine position for all DXA scans, with hands in the mid-prone position and palms facing inward for the whole-body scan, and arms crossed over the chest and legs fully extended using the manufacturer’s positioning aid for the lumbar spine and femur scans. A urine sample was collected to measure urine specific gravity (USG) using a handheld refractometer (Fisherbrand, Thermo Fisher Scientific, USA) to assess hydration status. A fasting blood sample was collected by venipuncture to measure concentrations of hemoglobin and TSH.

Maximal aerobic capacity (VO_2max_) was determined using a standardized graded treadmill protocol [23] and indirect calorimetry (TrueOne 2400, Parvo Medics, Murray, UT, USA). The test began with a 5-min warm-up at 0% gradient and a speed between 4.0 and 7.5 miles per hour (mph) based on the participant’s fitness level. Treadmill gradient was increased to 2.5% for one minute, followed by a self-selected increase in “workload” of either 0.5 mph or 2.5% gradient every minute thereafter until volitional exhaustion was reached. Rating of perceived exertion (RPE) was assessed during each stage using the Borg Scale (6–20). Oxygen uptake, respiratory exchange ratio (RER), and heart rate were collected in 15-s increments. To be considered a valid measurement of VO_2max_, participants had to achieve three of the four following criteria: (1) RER ≥ 1.10; (2) maximum heart rate within 10 beats of age-predicted maximum [208 – (0.7 × age)][24]; (3) evidence of a VO_2_ plateau defined ≤ 1.5 mL/kg/min change in VO_2_ with increased workload; (4) final RPE ≥18.

### Baseline Diet and Exercise

Habitual diet was assessed using food intake records collected on three consecutive days, including two weekdays and one weekend day. Participants were provided with written and verbal instructions for recording the time, location, quantity, and description of foods and beverages consumed (e.g., brand names, preparation methods, condiments), in addition to any dietary supplements. A printed packet of standardized food diagrams was also provided for reference. Food records were analyzed by a registered dietitian (TS) using Food Processor® Nutrient Analysis software (Version 11.9.14, ESHA Research, Salem, OR, USA). Typical exercise was tracked on the same three days using a GPS smartwatch (Venu SQ, Garmin International, Olathe, KS, USA), which measured heart rate, step count, exercise duration, and estimated energy expenditure.

### Experimental Conditions

#### Dietary Procedures

During the experimental conditions, participants were provided controlled, weighed diets equal to 30 kcal/kgFFM/d. Menus were designed by a registered dietitian using nutrient analysis software, Nutrition Data System of Research (NDSR 2022, University of Minnesota, Minneapolis, MN, USA). Diets consisted of three meals and one pre-run snack using whole foods and commercial products, and provided 55% of total energy from carbohydrates (CHO), 20% from protein, and 25% from fat. Two rotating menus were alternated daily (Menu A on Days 1, 3, and 5; Menu B on Days 2 and 4), containing animal-based sources of protein, including dairy (Supplemental Material A). Alternative menus without meat products (but including dairy) were available for vegetarian participants (n = 2). Participants received meals for a 24-h period and returned food packaging and uneaten food items, which were weighed to the nearest 0.5 g to assess actual intakes. Participants were instructed to consume meals at approximately the same time each day and to document consumption time on mealtime logs. Participants were instructed to consume the pre-run snack containing 30 g CHO one to two hours before the treadmill run to standardize CHO availability during exercise. Participants were instructed not to consume any other foods or beverages other than water and non-caloric beverages (e.g., black coffee, unsweetened tea, “diet soda”). A daily multivitamin (25 µg of vitamin D3 as cholecalciferol, 160 mg of calcium as calcium carbonate, 18 mg of iron as ferrous fumarate; Nature Made, West Hills, CA, USA) was provided to consume at a meal of their choice.

#### Running Procedures

On each day of the intervention, participants completed a supervised treadmill run between 65 and 70% VO_2max_. Running speed and duration were determined on the first day of the first experimental condition. VO_2_ was continuously collected during a controlled titration run, and speed was adjusted every four minutes until a steady-state VO_2_ of 65 to 70% VO_2max_ was achieved in the third and fourth minutes. The American College of Sports Medicine metabolic calculation [25] for running was used to predict starting running pace at 65% VO_2max_: V̇O_2_ (mL/kg/min) = 3.5 + (0.2 × S) + (0.9 × S × G), where S is speed in meters per minute (m/min) and G is gradient expressed as a decimal. Treadmill gradient was set to 1% to reflect the slightly higher oxygen cost when running outdoors [26]. Once the pace that elicited a 65–70% VO_2max_ was reached, the last two minutes of absolute VO_2_ (L/min) data were converted to kcal using a thermal equivalent of oxygen for the nonprotein respiratory quotient (RQ) of 0.9 (5.078 kcal/L) [27]. An RQ of 0.9 is equal to the nonprotein food quotient of the controlled diets (67.5% CHO, 32.5% fat).

On the first and last days of the experimental conditions, running economy or “efficiency” was measured at the start of the prescribed exercise bout. Participants completed three 4-min stages at 0% gradient, and heart rate was measured using a chest heart rate sensor (Polar H10, Polar Electro, Kempele, Finland). On Days 2–4, where no economy test was performed, runs began with an optional 5-min warm-up at 0% gradient. Daily run durations were adjusted to account for the energy expended during the titration run, economy tests (Days 1 and 5), optional warm-up (Days 2–4), and jumping exercises (RUN + J condition only), as applicable, to elicit the daily EEE of 15 kcal/kgFFM. Participants were instructed to refrain from physical activity outside of the supervised exercise sessions not related to activities of daily living (e.g., getting dressed, walking to the car).

#### Jumping Procedures

**Fig. 2:**
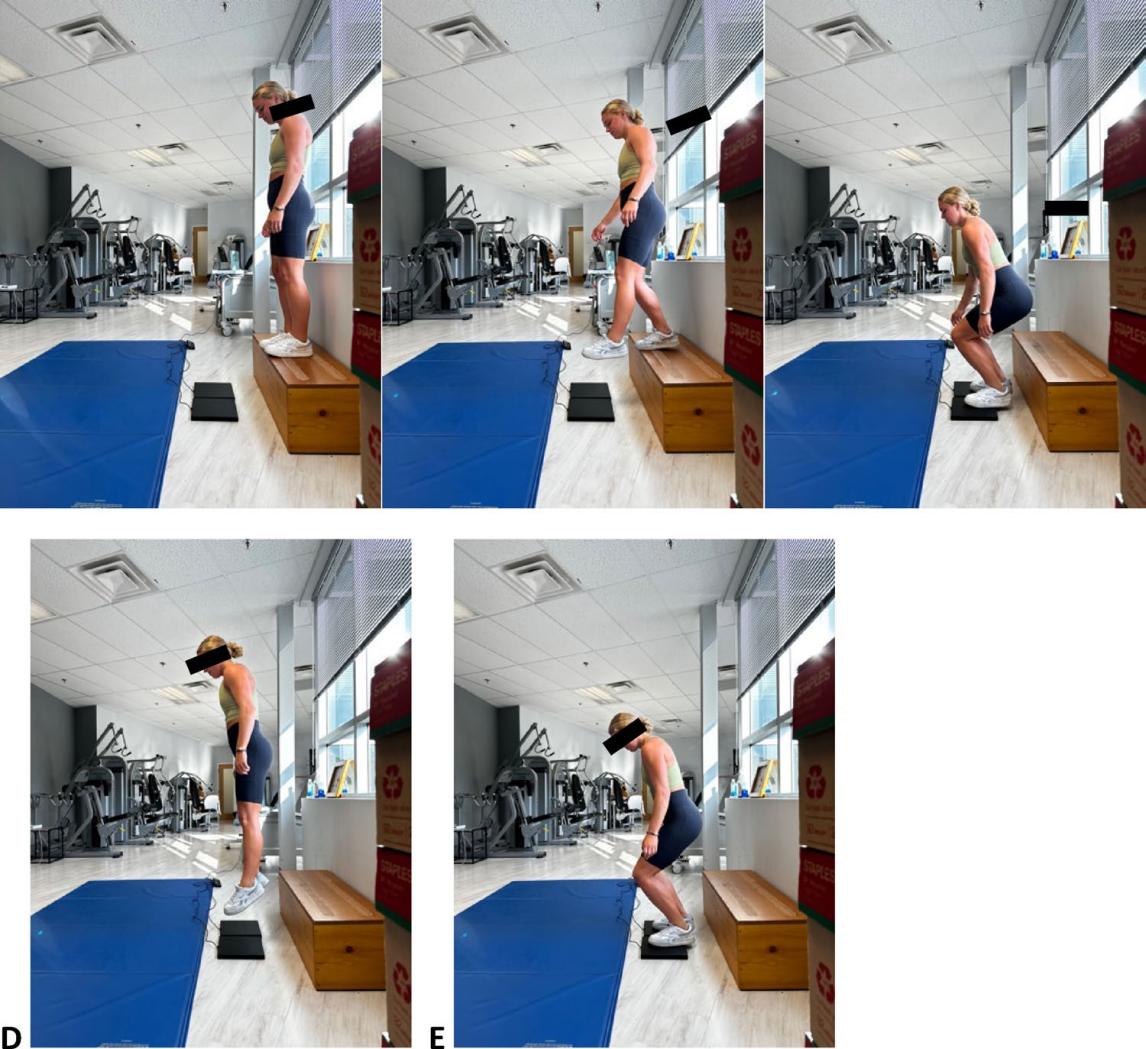
Description of the jumping procedure employed in the Running and Jumping Intervention (RUN + J) **A** Starting position was on top of a 30.5 cm box. **B** Participants stepped off the box and **C** landed evenly on both feet with knees bent to create momentum **D** for a fluid upward jumping movement and **E** final landing with knees slightly bent. This was repeated for five sets of 10 jumps with 60 s rest between each set

In the RUN + J condition, participants completed five sets of ten supervised depth jumps (50 total) from a height of 30.5 cm in the laboratory. The volume of impact exercise was based on the suggested exercise prescription for osteoporosis prevention [28]and longitudinal studies showing increased BMD with 50 daily jumps in premenopausal women [29, 30]. A description of the jump movement is presented in Fig. 2. Ground reaction force (GRF) was measured using NeuLog dual force plates (Eisco™ NeuLog™ Force Plate Logger Sensor NUL-225, Eisco Scientific, Victor, NY, USA). The intervention was designed to produce a GRF of at least two times body mass for each jump. A 60-s recovery period was taken between each set [28] during which participants sat or stood stationary. Energy expenditure of the jumping exercise was estimated on the first day of the RUN + J intervention using indirect calorimetry and assumed to remain consistent for the remainder of the intervention. Jumping exercises and the treadmill run were separated by at least four hours to allow for bone cell resensitization between loading bouts [31].

#### Blood Biochemistry

Blood samples were collected by venipuncture after an overnight, 10-h fast immediately before and after each intervention period to measure changes in markers of bone resorption (β-CTX), bone formation (P1NP, sclerostin), parathyroid hormone (PTH), reproductive hormones (progesterone and estradiol), thyroid hormones (free triiodothyronine [fT_3_], free thyroxine [fT_4_], and reverse T_3_ [rT_3_]), insulin, insulin-like growth factor (IGF-1), cortisol, and leptin. Total and free 25(OH)D concentrations were also assessed. Before centrifugation, serum specimens were clotted at room temperature (15 min to 2 h) and plasma specimens were kept on ice. Specimens were centrifuged at 3500 rpm for 13 min at 4 °C (Centrifuge 5804R 15amp, Eppendorf, Enfield, CT, USA). CBC, TSH, PTH, cortisol, and insulin were analyzed the same day at a commercial lab (LabCorp Burlington, Burlington, NC, USA). Aliquots of plasma and serum were stored at − 80 °C until batch analysis at the Virginia Tech Metabolism Core (Virginia Tech, Blacksburg, Virginia, USA). Single kit enzyme-linked immunosorbent assay (ELISA) was used to measure plasma P1NP (MyBioSource, San Diego, California, USA, intra-assay coefficient of variation (CV) ﻿ ≤ 5.05%) and rT_3_ (Alpco, Salem, NH, CV ≤ 8.1%), and serum β-CTX (EuroImmun, Mountain Lakes, NJ, CV ﻿ ≤ 5.29%), sclerostin (Thermo Fisher Scientific, Carlsbad, CA, CV ﻿ ≤ 1.96%), progesterone (Abcam, Cambridge, MA, CV ﻿ ≤ 10.4%), estradiol (Abcam, Cambridge, MA, CV ﻿ ≤ 7.67%), fT_3_ (Alpco, Salem, NH, CV ﻿ ≤ 3.6%), fT_4_ (Alpco, Salem, NH, CV ﻿ ≤ 4.26%), IGF-1 (Alpco, Salem, NH, CV ﻿ ≤ 5.83%), leptin (Alpco, Salem, NH, CV ≤ 5.5%), total 25(OH)D (Alpco, Salem, NH, CV ﻿ ≤ 3.2%) and free 25(OH)D (Alpco, Salem, NH, CV ﻿ ≤ 3.5%).

### Statistical Analysis

Statistical analyses were conducted in IBM® SPSS® Statistics software (Version 29.0.2.0 (14), IBM Corporation, Armonk, NY, USA) and ﻿graphs generated in Graphpad Prism (version 10, GraphPad Software, San Diego, CA, USA). Shapiro–Wilk tests were used to check for normality; data that were not normally distributed were log-transformed before analysis. No outliers were identified in the data based on visual inspection of box plots. One-sample t-tests were used to compare typical dietary intake and EA to the experimental LEA conditions, and to compare planned EA and EI to actual EA and EI after adjusting for food residuals. Paired t-tests were used to detect differences in total body mass and biochemistry between Day 0 of RUN and RUN + J. A linear mixed model was performed to assess outcomes for the impact of condition (RUN and RUN + J), time (Day 0 vs. Day 6), and condition by time interaction, with condition and time as fixed effects and participants as a random effect. Post hoc Bonferroni-adjusted paired t-tests or Wilcoxon-rank sum tests were conducted when main effects were statistically significant. The magnitude of change was determined by Cohen’s *d* effect size (﻿small ≥ 0.20, medium ≥ 0.50, large ≥ 0.80) [32]. Data are summarized as mean ± 1 standard deviation, and the level of significance was set at *P* < 0.05.

## Results

**Fig. 3:**
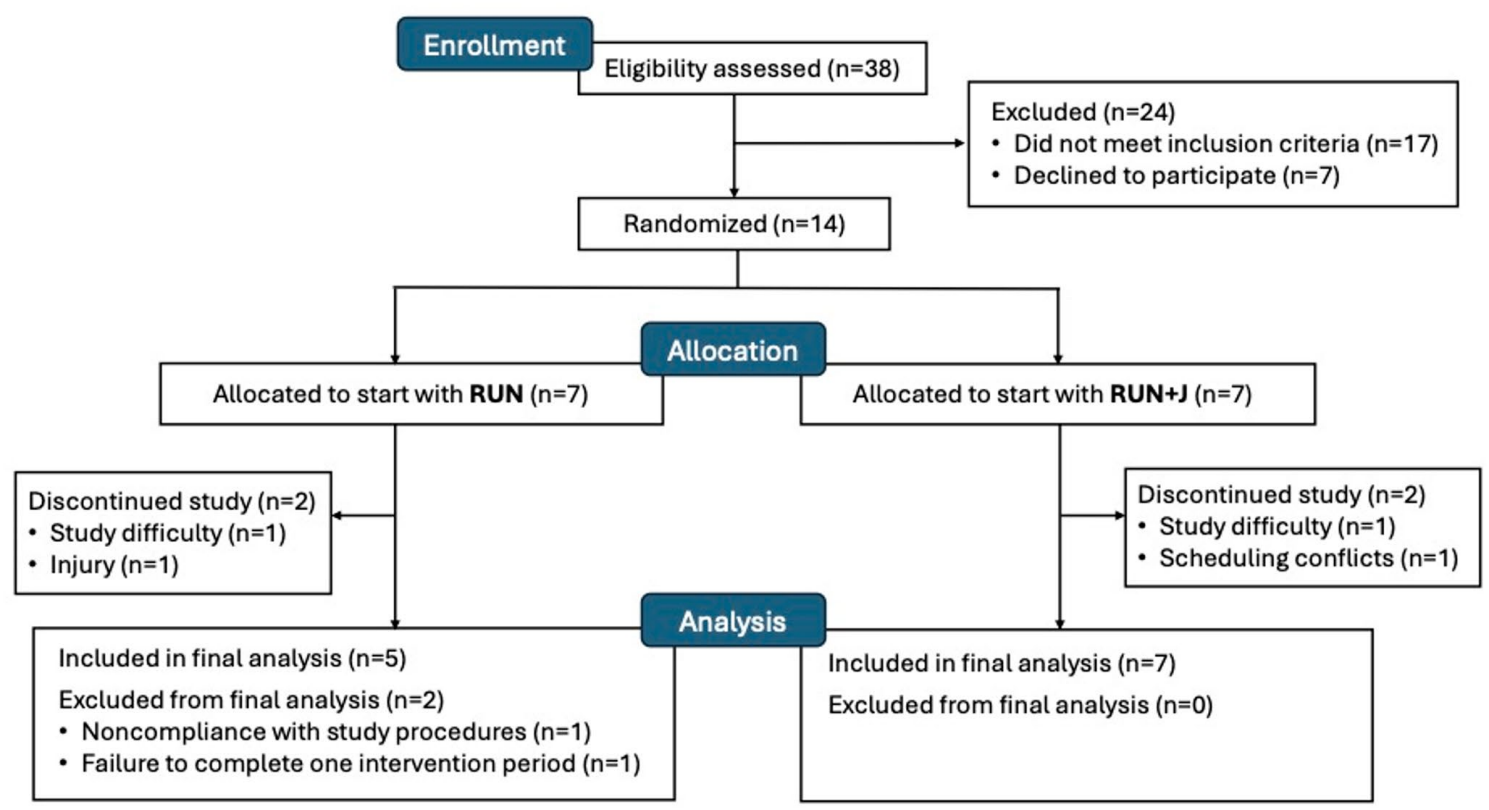
Enrollment flow diagram. Thirty-eight participants were screened initially for eligibility. Of the 38 screened, 17 did not meet the inclusion criteria and 7 declined to participate. Fourteen were randomized to the intervention, with seven allocated to start with the RUN condition and seven allocated to RUN + J. Four participants discontinued the study and two were excluded from the final analysis due to noncompliance with study procedures and failure to complete one full intervention period

Sixteen participants were initially recruited for the study. Fourteen participants started at least one experimental condition, thirteen completed at least one condition, and ten participants completed both conditions (Fig. 3). Seven participants were classified as Tier 1 (Recreationally Active) and seven as Tier 2 (Trained/Developmental) athletes. Seven participants each were allocated to start with the RUN and the RUN + J conditions. Two participants were excluded from the data analysis due to noncompliance with the exercise protocol (n = 1) and failure to complete one full intervention period (n = 1), resulting in 12 participants for the final analysis (Fig. 3). The baseline characteristics of these participants are summarized in Table 1.

**Table 1:** Baseline characteristics of study participants (n = 12)

Characteristics	Mean ± SD (range)
Age (year)	26.4 ± 3.9 (20.8–31.4)
Height (cm)	169.9 ± 5.5 (158.1–177.8)
Total Body Mass (kg)	66.6 ± 9.0 (48.8–80.1)
BMI (kg/m^2^)	23.1 ± 2.8 (19.7–29.9)
Fat Mass (kg)	18.8 ± 5.7 (11.2–28.4)
Body Fat (%)	28.8 ± 5.4 (21.4–38.8)
Lean Mass (kg)	45.1 ± 4.2 (35.5–50.1)
Fat-Free Mass (kg)	47.8 ± 4.5 (37.6–53.0)
BMC (g)	2646.3 ± 288.2 (2096.0–3100.0)
Lumbar BMD (g/cm^2^)	1.262 ± 0.171 (0.950–1.570)
Lumbar z-score	0.4 ± 1.2 (− 1.5–2.9)
Dual Femur BMD (g/cm^2^)	1.075 ± 0.101 (0.970–1.290)
Dual Femur z-score	0.4 ± 1.0 (− 1.5–2.2)
VO_2max_ (mL/kg/min)	45.3 ± 7.2 (37.0–55.6)
LEAF-Q score	3.5 ± 2.1 (1–7)
Hemoglobin (g/dL)	13.1 ± 0.6 (12.1–14.2)
TSH (µIU/mL)	1.67 ± 0.70 (0.97–3.32)

*BMI* body mass index, *BMC* bone mineral content, *BMD* bone mineral density, *LEAF-Q* Low Energy Availability in Females Questionnaire, *TSH* thyroid stimulating hormone

### Energy Intake and Exercise Energy Expenditure

Participants reported running 4.3 ± 1.3 days per week; five ran < 32 km/week, six between 32 and 48 km/week, and one between 48 and 64 km/week. Smartwatch measured EEE during baseline screening averaged 370 ± 160 kcal/day, which was less than the EEE of the LEA condition (*P* < 0.001). Baseline dietary intake compared to the diets provided in the LEA conditions is presented in Table 2. By design, absolute and relative EI and EA were lower in the LEA condition compared to baseline (*P* < 0.001 for all). The relative amounts of CHO (*P* = 0.048), protein (P = 0.004), and fat (*P* < 0.001) were also lower during the LEA conditions. Habitual macronutrient distribution was 46.0 ± 8.0% total energy from CHO, 18.0 ± 3.9% from protein, and 35.5 ± 4.5% from fat compared to the LEA conditions of 55% CHO, 20% protein, and 25% fat. The provided calcium during the experimental conditions was not different from normal intake (P = 0.755). After correction for food residuals, actual EI during RUN and RUN + J were 1388 ± 150 and 1383 ± 131 kcal. These intakes were not different from the EI target amount of 1436 kcal (RUN, *P* = 0.336; RUN + J, *P* = 0.191).

**Table 2:** Baseline dietary intake and exercise energy expenditure of participants compared to LEA conditions

Variable	Baseline	LEA Conditions	*P*-value
Energy Intake			
Absolute (kcal)	2101 ± 454	1436 ± 138	< 0.001
Relative (kcal/kgBM)	32.3 ± 8.9	21.7 ± 1.5	< 0.001
Exercise Energy Expenditure (kcal)	370 ± 160	712 ± 69	< 0.001
Energy Availability^a^ (kcal/kgFFM/day)	35.1 ± 9.9	15.0 ± 0.0	< 0.001
Carbohydrate			
Absolute (g)	246 ± 82	197 ± 19	0.064
Relative (g/kgBM)	3.8 ± 1.4	3.0 ± 0.2	0.048
Protein			
Absolute (g)	93 ± 18	72 ± 7	0.002
Relative (g/kgBM)	1.4 ± 0.4	1.1 ± 0.1	0.004
Fat			
Absolute (g)	83 ± 19	40 ± 4	< 0.001
Relative (% total kcal)	35.5 ± 4.5	25.0 ± 0.0	< 0.001
Calcium (mg)	836.7 ± 323.8	865.2 ± 82.9	0.755
Vitamin D (µg)	3.6 ± 6.0	25.2 ± 0.2	< 0.001
Iron (mg)	14.2 ± 5.5	32.1 ± 1.3	< 0.001

Baseline diet assessed using three-day food record analyzed by a registered dietitian. LEA conditions include daily multivitamin provided. Data reported as mean ± SD. *BM* body mass, *FFM* fat-free mass

^a^LEA calculated as (EI-EEE)/FFM

Day 0 of the RUN and RUN + J conditions began 3 ± 2 and 3 ± 1 days after the onset of menstruation (*P* = 0.460) with no differences in Day 0 serum estradiol (*P* = 0.120) or progesterone (*P* = 0.541) concentrations between conditions. Serum total 25(OH)D concentration was > 30 ng/mL for all participants on RUN Day 0 (53.0 ± 15.9 ng/mL; range 30.8–85.8 ng/mL), and for eleven of the twelve participants on RUN + J D0 (50.9 ± 12.0 ng/mL; range 28.7–71.2 ng/mL). There were no differences in total 25(OH)D or free 25 (OH)D concentrations on Day 0 between conditions (*P* = 0.505 and *P* = 0.900).

There were no differences in initial body weight between conditions. Reductions in total body mass were similar (*P* = 0.651), and averaged 1.3 ± 0.7 kg during RUN and 1.4 ± 0.7 kg during RUN + J.

To elicit an EEE of 15 kcal/kgFFM/d, participants ran between 56 and 82 min (70.8 ± 8.9 min) each day for a distance of approximately 10 km per session. Twelve participants completed the RUN + J intervention with 100% completion for the daily jumping exercises. Individual jump data is available in Supplemental Material B. The average GRF for all five days was 1972 ± 288 newtons (*N*), which was equivalent to a GRF of 3.0 ± 0.4 times TBM. GRF for all participants exceeded the target minimum GRF of two times TBM on all five days.

### Bone resorption and Formation Markers

**Fig. 4:**
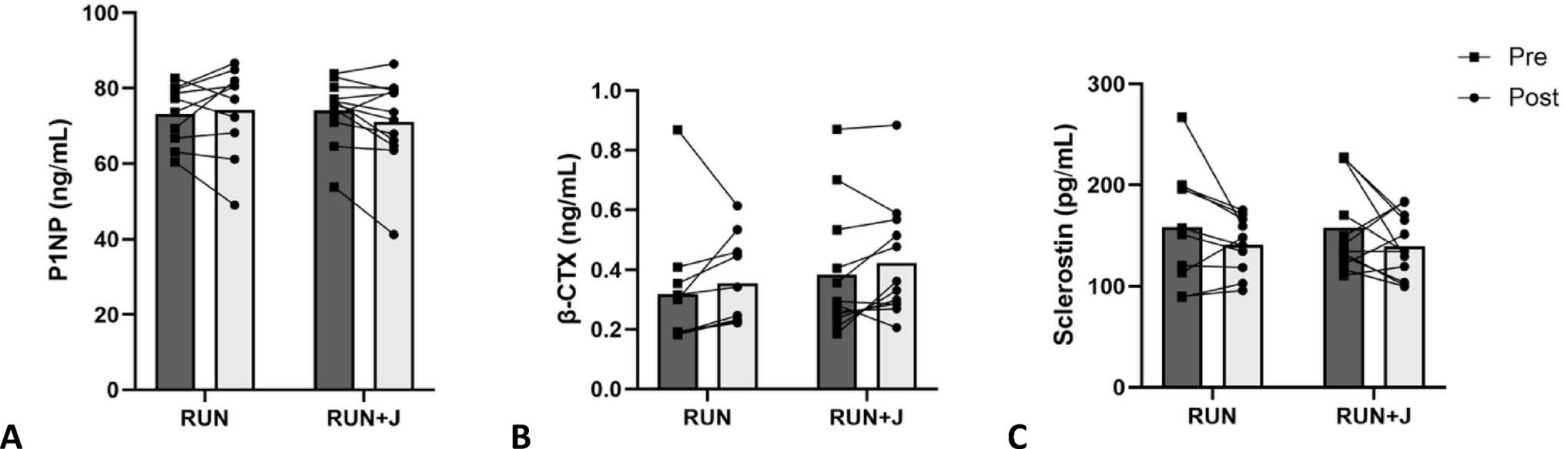
Individual changes in P1NP, β-CTX, and sclerostin during RUN and RUN + J intervention periods. Data presented as mean (bars) and individual changes (lines)

Bone resorption and formation markers are presented in Fig. 4 and summarized in Table 3. There were no significant main effects of time, condition, or time by condition interaction for P1NP or sclerostin. Baseline concentrations of β-CTX were higher during the RUN + J condition compared to the RUN condition (*P* = 0.004). Concentrations of β-CTX increased from Day 0 to Day 6 (main effect of time, *P* = 0.005) with no interaction effect between conditions (*P* = 0.716).

**Table 3:** Bone marker concentrations at Day 0 and Day 6 of RUN and RUN + J

	RUN (n = 10)		RUN + J (n = 12)		Time P-value	Condition P-value	Interaction P-value
	Day 0	Day 6	Day 0	Day 6			
P1NP (ng/mL)	73.1 ± 7.8	74.2 ± 11.9	74.1 ± 8.3	71.0 ± 11.9	0.517	0.558	0.187
β-CTX (ng/mL)	0.32 ± 0.21	0.36 ± 0.15	0.38 ± 0.21	0.42 ± 0.19	0.005	< 0.001	0.716
Sclerostin (pg/mL)	158.2 ± 57.2	141.2 ± 28.1	149.9 ± 39.2	139.7 ± 30.9	0.091	0.995	0.666

Data reported as mean ± SD. Abiations: P1NP, N-terminal propeptide of type 1 procollagen; β-CTX, ﻿C-terminal cross-linking telopeptide of type I collagenbrev

### Bone Regulatory Hormones and Growth Factors

Changes in endocrine markers of bone regulation and growth hormone concentrations are summarized in Table 4. Leptin was not normally distributed and was therefore log-transformed prior to analysis. Data summarized in Table 4 represent mean values before transformation.

**Table 4:** Hormones and growth factor concentrations at Day 0 and Day 6 in RUN and RUN + J

Variable	RUN		RUN + J		Time P-value	Condition P-value	Interaction P-value
	D0 (n = 10)	D6 (n = 10)	D0 (n = 12)	D6 (n = 12)			
PTH (pg/mL)	32.7 ± 10.7	31.7 ± 11.1	31.9 ± 10.5	35.3 ± 10.7	0.548	0.661	0.268
Free T_3_ (pg/mL)	3.4 ± 1.4	2.6 ± 0.9*	2.4 ± 1.0	2.1 ± 1.1	0.007	0.024	0.121
Free T_4_ (pg/mL)	1.49 ± 0.25	1.41 ± 0.10	1.36 ± 0.15	1.31 ± 0.15	0.040	0.004	0.492
rT_3_ (ng/mL)	0.58 ± 0.04	0.59 ± 0.03	0.57 ± 0.04	0.59 ± 0.03	0.020	0.771	0.451
IGF-1 (ng/mL)	88.3 ± 6.0	80.3 ± 6.4*	91.3 ± 10.5	79.8 ± 7.5*	< 0.001	0.249	0.338
Leptin (ng/mL)	8.5 ± 3.6	4.5 ± 2.3*	10.1 ± 4.9	5.2 ± 4.5*	< 0.001	0.745	0.357
Insulin (µIU/mL)	5.6 ± 2.9	4.0 ± 2.4	5.6 ± 2.2	3.8 ± 1.6	0.015	0.811	0.944
Cortisol (µg/dL)	17.0 ± 5.2	18.4 ± 4.0	17.6 ± 3.3	17.4 ± 4.8	0.606	0.920	0.477
Estradiol (pg/mL)	69.5 ± 19.4	70.8 ± 22.1	73.7 ± 21.8	71.9 ± 21.6	0.870	0.374	0.278

Data reported as mean ± SD. Abbreviations: PTH, parathyroid hormone; T_3_, triiodothyronine; T_4_, thyroxine; rT_3_, reverse ﻿triiodothyronine; IGF-1, insulin-like growth factor-1

*Significant from Day 0 in the same condition

All hormone concentrations on Day 0 were similar between conditions (*P* > 0.05) except for baseline fT_4,_ which was lower during RUN + J compared to RUN (*P* = 0.036) but still within normal reference range. Concentrations of fT_3_, IGF-1, leptin, and insulin decreased over time, but no significant interaction effect was detected. Concentrations of fT_3_ were 25% lower following the RUN condition (*P* = 0.022; *d* = 0.87) in post hoc analysis, but no change in fT_3_ concentration was shown following RUN + J (*P* = 0.237, *d* = 0.36).

## Discussion

This is the first study to investigate the effects of high-impact exercise and running on bone resorption and formation markers in female recreational runners under tightly controlled conditions of LEA. Contrary to our hypothesis, additional jumping exercises did not mediate changes in markers of bone resorption or formation in females exposed to conditions of energy restriction and high volume running.

In both LEA conditions, β-CTX increased over time without any change in P1NP, which contradicts previous studies showing suppressed P1NP during short-term LEA [17, 33]. It is possible that the suppression of bone formation previously shown during periods of LEA are dependent on the type and volume of exercise used to induce LEA. For example, completing 20 high-impact jumping exercises twice daily without additional exercise did not prevent reductions in P1NP following three days of LEA (15 kcal/kgFFM/d) in active women [17]. Conversely, participants in the present study ran continuously for approximately 70 min/day covering a distance of approximately 10 km per session, which exceeded the reported habitual weekly running volume of most participants. It is plausible that the β-CTX and P1NP patterns observed were reflective of early-stage bone adaptation to the exercise stimulus as opposed to the LEA condition alone. An increase in β-CTX would be expected in response to progressive training as remodeling sites are established, and it would take several weeks or months for a rise in P1NP to be observed during the bone formation phase [34]. This is in partial agreement with a study of active women with similar baseline fitness (VO_2_ peak = 47.9 ± 5.5 ml/kg/min; habitual EEE = 402 ± 227 kcal/d) and experimental training volume (66 ± 4 min/d running at 70% VO_2_ peak), which found an increase in β-CTX in response to a 5-day LEA condition (15 kcal/kgFFM/d) [33]. However, they also reported a significant decrease in P1NP concentration which was not observed in the present study. Conversely, Papageorgiou et al. [16] found no change in P1NP or β-CTX in active women exposed to three days of LEA (15 kcal/kgFFM/d) where participants completed high volume running that exceed habitual training. The discrepancy in these results may be partially attributed to differences in study duration. Although β-CTX responds acutely to stimuli, such as food intake and exercise [35], it may take several days to detect changes in resting concentrations of active individuals. This could partially explain why β-CTX was shown to increase in the current study but not in similar three-day investigations.

Sclerostin has been shown to increase in response to LEA (15 kcal/kgFFM/d) without exercise [36] but not following the same level of LEA with moderate-intensity running [33]. Although there was no significant effect of either LEA condition on sclerostin, there was a trend towards reduced concentrations over time. Sclerostin is a protein secreted by osteocytes that downregulates bone formation by inhibiting the Wnt/β‐catenin pathway and osteoblasts synthesis [37]. A reduction in sclerostin concentration may indicate the beginning of bone reversal in the remodeling cycle that would lead to subsequent bone formation. It is possible that changes in P1NP may have been observed with a longer intervention or follow-up period. There may have also been different outcomes in runners who were already accustomed to the higher training volume, as the running protocol would not have been a novel stimulus for bone remodeling.

It is possible that the jumping protocol did not provide enough of a stimulus to elicit positive osteogenic effects in the presence of LEA. In a previous study where brief jumping exercises were shown to successfully prevent LEA-induced increases in bone resorption markers [17], the exercise protocol was multidirectional and produced a higher GRF than the present study. Since running already provides a high-impact stimulus to the lower limbs, we may not have observed any positive effects based on our unidirectional protocol that only targeted one region of bone. A greater osteogenic response may have been elicited by exercises targeting the trunk or upper body. The lack of differences in bone markers between conditions also suggests that engaging in brief, high-impact exercise in addition to normal training does not pose an increased risk to bone health in recreational runners. This is significant given the potential beneficial effect of cross-training on BMD in recreational runners [38].

Reduced concentrations of fT_3_, IGF-1, leptin, and insulin, and increased rT_3_ concentration following the LEA conditions reflect similar changes reported in short-term LEA trials [10, 16, 33, 39]. These data indicate participants were adherent to the dietary protocol and in a state of energy deficiency. The additional jumping exercises did not affect the response of IGF-1, leptin, insulin, or rT_3_ to the LEA exposure. However, concentrations of fT_3_ were only significantly decreased following the RUN condition. Previous studies have reported decreased total T_3_ in women following three days of diet-induced LEA (15 kcal/kgFFM/d), but not during LEA when participants are either running [16] or performing jumping exercises [17]. More research is needed to understand the variability in T_3_ response to LEA with high-impact exercise.

Bone loss associated with estrogen deficiency has been shown in young amenorrheic endurance athletes [40] and postmenopausal elite runners not on hormone replacement therapy [41]. Estradiol regulates bone resorption by promoting the expression of osteoprotegerin, a decoy receptor for receptor activator of nuclear factor-kappa β ligand (RANKL), and downregulating osteoclast activity [42]. In the present study, no changes in resting estradiol concentrations were shown following either condition, which is in agreement with previous studies utilizing the same level of EA [16, 17, 33]. In contrast, 24-h mean estradiol concentrations were shown to decrease by 15% after five days of LEA equal to 10 kcal/kgFFM/d [10]. It is possible that estradiol concentrations are not influenced until EA drops below 15 kcal/kgFFM/d, which may partially explain why changes in estradiol concentration were not detected in the present and previous investigations.

Despite the tightly controlled nature of this study, our end sample size and missing data from two participants may have limited our ability to detect differences in hormone concentrations between conditions. Mean VO_2max_ in the present study was similar to previous investigations in recreational female runners [16, 33] with a broad range amongst participants. The variance in VO_2max_ likely reflects the broader population of recreational runners but may have affected individual responses to the treatment and has limited generalizability to highly trained runners. Participants were also permitted to engage in their normal activities of daily living and may not have been completely sedentary during the 4-h recovery period between the running and jumping exercises. Not restricting their movements between exercise sessions strengthened the ecological validity of this study, but it may have hindered bone resensitization and response to the second bout of exercise. It should also be acknowledged that measurements of P1NP and β-CTX reflect the synthesis and degradation of type 1 collagen, which is not specific only to bone tissue [15]. Thus, it cannot be determined if the changes in P1NP and β-CTX are solely reflective of changes in bone tissue or bone (re)modeling at a particular skeletal site. The laboratory technique for measuring primary bone outcomes was also limited to ELISA, which is an accurate assay method but has lower precision than the automated electrochemiluminescence immunoassay (ECLIA) technique. Finally, it is possible that short-duration LEA studies, such as the present one, do not find changes in P1NP because bone resorption precedes bone formation in the remodeling cycle. Follow-up blood samples were only collected on the morning immediately after each study condition ended and may have missed delayed changes that would influence the interpretation of our findings.

In conclusion, our results indicated that high-impact jumping exercises, in the presence of high volume running, did not have an effect on short-term changes in bone marker concentrations during LEA in female recreational runners. These results also suggest the addition of high-impact exercise during LEA does not exacerbate the effect of exercise and energy restriction on bone markers.

## Supplementary Information

Below is the link to the electronic supplementary material.

Supplementary Material 1.

## Data Availability

Data supporting the findings of this study are available within the paper and its Supplementary files. Raw data are available upon reasonable request from the corresponding author.
