# Supplementary material for: Effects of High-Impact Exercise on Bone Marker Concentrations During Controlled Low Energy Availability in Recreational Female Runners
Source: Calcif Tissue Int. 2026 Jun 25;117(1):101. doi: 10.1007/s00223-026-01564-0 (PMC13303319; doi:10.1007/s00223-026-01564-0)
Supplement: Supplementary file 1 — Supplementary Material 1. [file 223_2026_1564_MOESM1_ESM.docx]

Effects of additional high-impact exercise on bone marker concentrations during controlled low energy availability in female recreational runners

Trisha Sterringer^1^, Craig Sale^2^, Anna Morozov^1^, Jiarui Liang^1^, D. Enette Larson-Meyer^1^

1. Virginia Tech, Blacksburg, VA, USA

2. Manchester Metropolitan University, Manchester, UK

Corresponding author: [tsterringer@vt.edu](mailto:tsterringer@vt.edu)

ORCID:

Trisha Sterringer: 0000-0001-7959-9412

Craig Sale: 0000-0002-5816-4169

D. Enette Larson-Meyer: 0000-0001-9695-5474

Supplemental Material A

| **Meal** | **Menu A** | **Menu B** |
| --- | --- | --- |
| **Breakfast** | - Low-sugar instant oatmeal - Nonfat plain Greek yogurt with blueberries and granola | - Whole wheat bagel with cream cheese - Nonfat plain Greek yogurt with blueberries and granola |
| **Lunch** | - Salad: Quinoa, lettuce, red cabbage, carrots, slivered almonds, sesame ginger dressing - Wheat crackers | - Salad: Brown rice, lettuce, cucumber, red pepper, feta cheese, low-fat Greek dressing - Wheat crackers |
| **Pre-Run Snack**  *1-2 h before run* | - Pretzels (30g CHO) | - Pretzels (30g CHO) |
| **Dinner** | - Commercial frozen dinner with chicken - Whole wheat bread and butter - Dark chocolate square | - Commercial frozen dinner with beef - Whole wheat bread and butter - Dark chocolate square |

**Example of rotating menus for controlled diets.** Menu A consumed on Days 1, 3 and 5. Menu B consumed on Days 2 and 4. Menus provided 30 kcal/kgFFM/d with 55% total kcal from carbohydrate (CHO), 20% from protein, and 25% from fat. Food items adjusted as necessary based on individual preferences to increase adherence.

Supplemental Material B

**Daily ground reaction force (GRF) for depth jumps.** Individualized GRF target is represented by the black bars and the measured daily average GRF in the colored bars.

Supplemental Material C

|  | Baseline screening (n=11) | RUN (n=8) | RUN+J (n=11) |
| --- | --- | --- | --- |
| Resting HR (bpm) | 54.0±6.0 | 51.2±6.8 | 51.3±5.7 |
| Step count | 12,217±4342 | 17,806±2607 | 17,979±2472 |
| Activity (min/day) | 42.8±22.2 | 83.3±19.0 | 79.8±27.9 |

**Smartwatch data for average resting heart rate, active minutes, and step count at baseline and during the intervention periods.** Data is missing for some participants due to smartwatch malfunction (baseline, n=1; RUN, n=2; RUN+J, n=1). Data is summarized as mean ± standard deviation.

Abbreviations: HR, heart rate; bpm, beats per minute; min, minutes.
